# Supplementary material for: Is there a relationship between isometric hamstrings-to-quadriceps torque ratio and athletes’ plyometric performance?
Source: PLoS One. 2023 Nov 16;18(11):e0294274. doi: 10.1371/journal.pone.0294274 (PMC10653514; doi:10.1371/journal.pone.0294274)
Supplement: Attachment — Submitted filename: response to the comment1.docx [file pone.0294274.s002.docx]

**Responses to the comments**

We very much appreciated your encouraging and insightful comments. We have endeavored to respond to all suggestions and comments, which further improved the understanding and potential impact of our paper. We responded to the mentioned comments in both the “revised manuscript” file and this one. In the manuscript, responses to the first reviewer have been highlighted green and yellow for the second reviewer. Hope our effort meets the editorial board's expectations.

Sincerely Yours,

Authors.

***Reviewer: 1***

First of all, thank you for valuable effort on this manuscript.

**Authors:** Dear Professor, thank you for your time and consideration.

The following points are presented after my review.

- The title of the study should be revised. The entire text has been academic language-reviewed.

**Authors:** Done.

- Is "double" meant to be denoted by "dabble" in the term "dibble leg-drop vertical jump"? The use of the word "dabble" does not seem appropriate.

**Authors:** It was a typo. It's corrected and highlighted in that manuscript.

- It is recommended to briefly indicate which parameters are used in the RSImod calculation in the method section of the summary.

**Authors:** Done.

- The H:Q ratio mentioned in the Introduction section is evaluated as isometric and more recently isokinetic tests (because it provides the opportunity to perform functional analysis). Within the scope of this study, the reasons why the isometric H:Q ratio will be examined instead of the dynamic H:Q ratio should be given in more detail.

**Authors:** Dear Professor, you are right, an isokinetic test could have been used to calculate the dynamic strength ratio; But, we considered the accessibility, portability, and more clinically applicable of the device!! And the validity and reliability of HHD were also approved in various research; These reasons are mentioned and highlighted in the manuscript. Although, not using a dynamics ratio by Isokinetic device was mentioned as a study limitation in the manuscript.

- Although it is mentioned for what purpose the RSDmod index is used or what it is an indicator of, it has not been mentioned exactly how it is calculated. For a first-time reader, although methods are also mentioned, it would be better to include a brief explanation over a task in this section, at least by mentioning which physical parameters it is calculated using for this index.

**Authors:** Dear Reviewer, it is briefly mentioned and highlighted in the introduction section; And of course, It was mentioned with more details in the methods section.

- "If yes! to what extent does increasing or decreasing H:Q torque ratio affect the athletes' jumping performance?" Instead of this question, a interpretation can be made based on the according results, or not specified at all.

**Authors:** Good comment. It is revised and highlighted in the manuscript.

- Despite the power analysis, sample size is limited. Considering the inclusion of subjects of different genders, the number of subjects is low compared to other similar studies (usually single-gender subjects were evaluated in other similar studies) and in terms of making a clear conclusion from the results of the study.

**Authors:** Dear Reviewer, all of the participants were male in this study. It is mentioned and highlighted in the manuscript.

- No information on gender distribution was provided.

**Authors:** Dear review, as mentioned in the manuscript, all of the subjects in this study were recreational male athletes. It's highlighted in that manuscript.

- It is recommended to mention which sports the subjects are doing.

**Authors:** Done.

- Ethics committee approval protocol number should be attached.

**Authors:** Done.

- If the tests were done in a single session, it should be stated whether sufficient rest breaks are given between muscle test and performance tests and how long it is given.

**Authors:** Yes, it was a single-session test and, of course, a 5 min rest was given to athletes between muscle test and performance tests. It's mentioned and highlighted in that manuscript.

- As a procedure explanation, which test has been done first, the procedure of this test should be given first, followed by the other. The relevant sections should be rearranged by paying attention to the order of the test procedure explanations. A subtitle can be created about jump tests, as others.

**Authors:** Good comment. Done.

- The figures in figure 1 are illustrative but it has not clearly seen as one leg during landing on the SL-DVJ task. Is it the first landing on one leg or the second landing? or both of them? It is recommended to review and revise the figure from this perspective.

**Authors:** Dear Professor, actually, the athletes were asked to perform the SL-DVJ task with the dominant leg! So both landings were done on one leg. The figure is revised.

- Under the heading "The hamstring-to-quadriceps (H:Q) torque ratio measurement", the calculation part should be placed after the test procedure, not at the beginning of the paragraph.

**Authors:** You are right. It is revised and highlighted in the manuscript.

- The quadriceps femoris muscle, generally referred to as "quadriceps" throughout the manuscript, should be corrected.

**Authors:** Done.

- The relationship between isokinetic H/Q ratio and jumping performance parameters was investigated without using RSDmod. Some recent studies are listed below. The discussion can be rearranged by reviewing these studies.

o Atik, B., Ayberk, B., Özgül, B., & Polat, M. G. (2023). The Association Between Isokinetic Strength and Strength Asymmetry and Jump Performance in Female Volleyball Players. Sport Sciences for Health, 1-8.

o Schons P, Da Rosa RG, Fischer G et al (2019) The relationship between strength asymmetries and jumping performance in professional volleyball players. Sports Biomech. 18(5):515–526.

**Authors:** The discussion section is revised.

- One of the evaluations applied in the study is a static measurement while the other is a dynamic task. Therefore, instead of a static measurement, comments can be added about the reflection of dynamic muscle strength (isokinetic) evaluations on RSDmod can be examined. Although this situation is partially mentioned at the end of the discussion, comparative comments can be made in the first parts of the discussion by giving place to case studies from the literature.

**Authors:** Of course, it is mentioned and highlighted in the manuscript.

- It should be noted that RSDmod was not used in the studies mentioned in sources 23 and 24.

**Authors:** You are right. It is mentioned and highlighted in the manuscript.

- Since the RSDmod index was not used in the mentioned studies, "According to the finding of the current study and considering the results of previous studies, the authors hypothesize that as much as the complexity of the movement is increase, the correlation between H:Q torque ratio and RSImod decreases " can be commented, it is recommended to review again.

**Authors:** Good comment**.** It is revised and highlighted in the manuscript.

- The results of the study may also have been affected by the evaluation of athletes doing different sports.

**Authors:** Good comment. It is added to the study limitation.

- The discussion generally seems rather inadequate. It should be rearranged by including current similar studies.

**Authors:** Done.

***Reviewer: 2***

Reviewer #2: The subject raised by the authors is extremely interesting. The effect of the H/Q ratio on the risk of injury is quite extensively described. However, there are few studies describing the relationship between the H/Q ratio and athletes performance. Therefore, the presented manuscript is part of filling the current knowledge gap.

The first chapter adequately introduces the reader to the problem and shows the gap in knowledge to be filled.

**Authors:** Dear Professor, thank you for your time and consideration.

The methods have been described quite extensively. However, authors should use a different abbreviation than SJ for "stop jump". The abbreviation SJ is adopted and used for squat jump. Therefore, one should not suggest to the reader that the article concerns a different, biomechanically standardized jump.

**Authors:** You are right. It is revised and highlighted in the manuscript.

The results were described and presented in sufficient detail. However, specifying the RSI unit as m/s should be considered overzealous and unnecessary. We don't want to talk about movement velocity here.

**Authors:** Dear professor, you are right and we didn’t talk about movement velocity in the other part of the article; Actually, the unit of RSI was just mentioned in the table.

In the Discussion - I couldn't find an author named Dekker [24]... I think it was Diker, right?

**Authors:** You are right. It is revised and highlighted in the manuscript.

The interpretation of the obtained results and the final conclusions seem to be correct.

**Authors:** Thank you.
