# Supplementary material for: Is there a relationship between isometric hamstrings-to-quadriceps torque ratio and athletes’ plyometric performance?
Source: PLoS One. 2023 Nov 16;18(11):e0294274. doi: 10.1371/journal.pone.0294274 (PMC10653514; doi:10.1371/journal.pone.0294274)
Supplement: Attachment — Submitted filename: response to the comment1.docx [file pone.0294274.s003.docx]

**Responses to the comments**

We very much appreciated your encouraging and insightful comments. We have endeavored to respond to all suggestions and comments, which further improved the understanding and potential impact of our paper. We responded to the mentioned comments in both the “revised manuscript” file and this one. In the manuscript, responses to the first reviewer have been highlighted green and yellow for the second reviewer. Hope our effort meets the editorial board's expectations.

Sincerely Yours,

Authors.

***Reviewer: 1***

Firstly thank you for your effort on the revisions of the manuscript. With the revisions made, it is now in a better condition.I would only like to emphasize the points I have stated in the following two items and recommend that the necessary revisions be made.

**Authors:** Dear Professor, thank you for your time and consideration.

1. It seems that the word "double" should be used throughout the text instead of "dabble", as used in the summary.

**Authors:** Done.

2. Although it is noted that the necessary revisions have been made and highlighted in the text regarding the comments below, when the initial version and the revised version of the manuscript are compared, the sections highlighted in green and stated to be revised are exactly the same as the sections in the initial version. I guess there was an error in revising the relevant sections. I recommend that it be reviewed and the information obtained from the suggested current references mentioned in the reviewer comments be added to the discussion section.

**Authors:** Dear Professor, thank you for your consideration. By using the mentioned references, the discussion section is revised. But, we added this new comment in the last paragraph of the discussion; Adding to the initial part of the discussion was a bit difficult.

***Reviewer: 2***

All my comments were completed properly. I am satisfied with the corrections.

I would like to thank the authors for their work.

**Authors:** Dear Professor, thank you for your time and consideration.
